# Lipophilicity Determination of Quaternary (Fluoro)Quinolones by Chromatographic and Theoretical Approaches

**DOI:** 10.3390/ijms20215288

**Published:** 2019-10-24

**Authors:** Krzesimir Ciura, Joanna Fedorowicz, Filip Andrić, Katarzyna Ewa Greber, Alina Gurgielewicz, Wiesław Sawicki, Jarosław Sączewski

**Affiliations:** 1 Department of Physical Chemistry, Faculty of Pharmacy, Medical University of Gdańsk, Al. Gen. J. Hallera 107, 80-416 Gdańsk, Poland; greber@gumed.edu.pl (K.E.G.); alina.gurgielewicz@gmail.com (A.G.); wsawicki@gumed.edu.pl (W.S.); 2 Department of Chemical Technology of Drugs, Faculty of Pharmacy, Medical University of Gdańsk, Al. Gen. J. Hallera 107, 80-416 Gdańsk, Poland; jfedorowicz@gumed.edu.pl; 3 Department for Analytical Chemistry, University of Belgrade-Faculty of Chemistry, Studentski trg 12–16, 11000 Belgrade, Serbia; andric@chem.bg.ac.rs; 4 Department of Organic Chemistry, Faculty of Pharmacy, Medical University of Gdańsk, Al. Gen. J. Hallera 107, 80-416 Gdańsk, Poland; jaroslaw.saczewski@gumed.edu.pl

**Keywords:** lipophilicity, micellar electrokinetic chromatography, thin-layer chromatography, *Safirinium* hybrids

## Abstract

Lipophilicity is a vital physicochemical parameter of a molecule, which affects several biological processes such as absorption, tissue distribution, and pharmacokinetic properties. In this study, evaluation of lipophilicities of a series of novel fluoroquinolone-*Safirinium* dye hybrids using chromatographic and computational methods is presented. Fluoroquinolone-*Safirinium* dye hybrids have been synthesized as new dual-acting hydrophilic antibacterial agents. Reversed phase thin-layer chromatography and micellar electrokinetic chromatography experiments were carried out. Furthermore, log*P* values of the target structures were predicted by means of different software platforms and algorithms. In order to assess similarities and dissimilarities of the obtained lipophilicity indexes, cluster analysis and sum of ranking differences were performed. The significant differences of calculated log*P* values (α = 0.05, *p* < 0.001) indicated that an experimental approach is necessary for lipophilicity prediction of this class of antibiotics. Chromatographic data indicated that the newly synthesized hybrid (fluoro)quinolone-based quaternary ammonium derivatives show less lipophilic character than the parent (fluoro)quinolones. Additionally, the chromatographically obtained lipophilicity indexes were evaluated for possible application in quantitative retention–activity relationships. The established lipophilicity models have the potential to predict antimicrobial activities of a series of quaternary (fluoro)quinolones against *Bacillus subtilis, Escherichia coli,* and *Proteus vulgaris.*

## 1. Introduction

Lipophilicity is one of the most frequently examined physicochemical properties of drug candidates. Typically, it is determined in order to support quantitative structure-activity relationships (QSAR), including prediction of biological process such as absorption, tissue distribution, and others pharmacokinetic properties [1,2]. Moreover, lipophilicity is also taken into account in lipophilic ligand efficiency assessments (LLE) [1,3].

Lipophilicity is characterized by solute distribution in biphasic liquid-liquid or solid-liquid systems. The traditional method proposed by Hansch and co-workers involves a shake-flask procedure where partition coefficient of the target compound between *n*-octanol and water (log*P*) is assessed. Although this method is practically not applied nowadays, the log*P* universal scale is generally used to represent lipophilic character of a molecule [4].

Currently, three methods for lipophilicity assessment are recommended by the Organization of Economic Co-operation and Development: shake-flask, high-performance liquid chromatography (HPLC), and slow-stirring [4].

Among these methods chromatographic approaches are very widespread, since such indirect methods demonstrate significant advantages compared to a shake-flask procedure. The chromatographic approach is inexpensive, rapid, and requires a small amount of substances that do not need to be very pure, as their impurities are readily separated during the chromatographic analysis [5]. What is more, the chromatographic methods are repeatable and robust. It should be emphasized that the methods based on the solid-liquid partitioning are very convenient in early steps of drug development when a high-throughput is required instead of a high accuracy.

Other indirect methods based on electrokinetic chromatography (EKC) have been proposed as alternatives for lipophilicity estimation, including micellar electrokinetic chromatography (MEKC) and microemulsion electrokinetic chromatography (MEEKC). 

Furthermore, the in silico approach has been proposed for lipophilicity assessment. Recently, several software programs for log*P* calculation have been developed. Nevertheless, it should be emphasized that experimental data are still preferred. Hence, significant differences between the calculated log*P* values for the same chemical structures using various theoretical approaches can be regarded as one of the major limitations of the computational methods. These discrepancies can be explained by the fact that newly synthesized drug candidates may contain substructures or heterocyclic systems that are not covered by the software development training set [2]. Other limitations may include misclassification of some potentially active compounds in terms of their log*P* value based on the Lipinski’s rule of 5 [3,6].

A series of novel fluoroquinolone-*Safirinium* dye hybrids have been synthesized as new dual-acting hydrophilic antibacterial agents [7]. These conjugates cause perturbation of the lipid bilayer of the bacterial cytoplasmic membrane and the outer membrane of gram-negative bacteria due to the presence of quaternary ammonium group, and inhibition of DNA gyrase/bacterial topoisomerase IV elicited by fluoroquinolone portion. 

The main goal of this study was to evaluate the lipophilicities of a series of fluoroquinolone-*Safirinium* dye hybrids and their parent fluoroquinolones using typical reversed phase thin layer chromatography (RP-TLC) approach, MEKC, and computational methods. In order to compare log*P* values calculated by various algorithms, cluster analysis (HCA) was performed. The obtained lipophilicity parameters were correspondingly grouped by the nonparametric method, based on sum of ranking differences protocol. Furthermore, the chromatographically obtained lipophilicity indexes were evaluated for possible application in quantitative retention–activity relationships (QRAR). The proposed QRAR equations can be used for prediction of antimicrobial activities of this chemical group of antibiotics. 

## 2. Results and Discussion

### 2.1. Computationally Estimated Lipophilicity Parameters

The computational approaches for lipophilicity determination have several advantages over the experimental methods, including short calculation time. Another benefit is the fact that calculated log*P* parameters can be obtained prior to synthesis. Consequently, the first selection of structures with desired lipophilicity can be done when the drug candidates are designed. Hence, computational methods can save time and chemical reagents. For these reasons, the computational approaches are very attractive from the economic and environmental points of view. 

Log*P* values for all the investigated compounds predicted by means of 10 different software platforms and algorithms are summarized in Table 1. 

**Figure 1:**
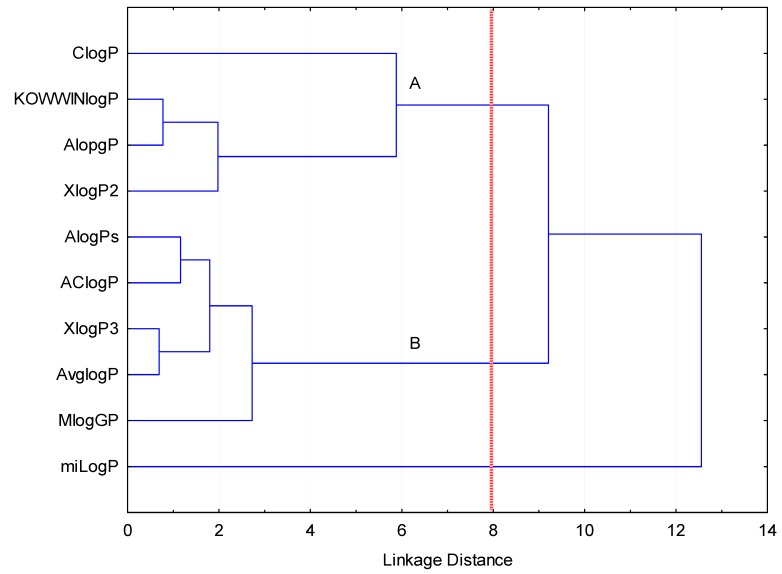
Results of cluster analysis (HCA) analysis of in silico estimated log*P* values. At eight distance units two clusters of interest can be observed (**A** and **B**), and one outlier (**miLog*P***).

The algorithms utilized are based on various theoretical methodologies. Briefly, there are three main groups of algorithms used for log*P* calculations: Atomic approach, fragment contribution technique, and properties-dependent methods. Classification of the investigated software based on algorithm type is presented in Table 2. HCA was performed in order to show similarities and dissimilarities between the log*P* values calculated with various programs. Among the agglomerative clustering methods, Ward’s method was selected because of its unique properties. It is based on a classical sum-of-squares criterion, producing groups that minimize within-group dispersion at each binary fusion [8]. Results of CA analysis are presented as a tree diagram in Figure 1. 

The simplest theoretical models are established on pure atomic approaches, which presuppose that each atom contributes to the log*P* parameter (AClog*P*). More complex algorithms are implemented into Xlog*P*2 since this atom-based method is corrected by factors integrated within the program. In case of Xlog*P*3, calculations start from similar reference structures with known log*P*, which are corrected with the atomic approach. The results obtained with approaches that use the atomic-based methods are predominantly grouped in the second cluster (B). One would expect that software which utilizes the fragment contribution should give more accurate results. The advantage of this calculation method compared with the atomic approach is the fact that this method includes information of electronic or intramolecular interactions. However, HCA found that ChemDraw and miLog are outliers. Among the tested programs, Alog*P* and Mlog*P* employ property-dependent algorithms. Alog*P* is found in cluster A together with KOWWINlog*P,* which utilizes hybrid algorithm combining both an atom-based approach and fragmental contribution method. Although Mlog*P* applies topological indexes, it is found in the cluster B, together with AClog*P*, Xlog*P*3, and average log*P*. Alog*P*s use a self-learning method based on associative neuronal networks from molecular structures. It is grouped with the AClog*P*, Xlog*P*3, and average log*P* in the cluster B. 

The calculated log*P* values for the newly synthesized quinolone derivatives (whose structures are presented in Table S1) reveal huge differences. For example, miLog*P* approach results in −5.08 for compound 1, while Xlog*P*3 scheme provides a notably contradictory value of 3.57. These differences can be to some extent explained by the presence of quaternary ammonium cation in structures of the newly synthesized quinolone derivatives. Hence, these hybrids are permanently charged, which can cause difficulties in lipophilicity calculation. 

Nevertheless, a similar situation takes place when comparing log*P* values calculated for parent (fluoro)quinolone antibiotics (FQs), for example ciprofloxacin (milogP −3.57 vs XlogP2 1.46). The same structures could be hydrophilic or lipophilic depending on the software applied. The estimated log*P* values with atom-based methods are higher compared to other methods, except for Alog*P* parameter. Similar observations have been noticed by Kłosińska-Szmurło and co-workers for FQs antibiotics [9]. These findings confirm the thesis that correction of the calculated log*P* values is necessary. Steric and electronic interactions can significantly influence lipophilicity of this class of compounds. Summing up, the observed huge differences among the calculated log*P* values clearly demonstrate that the experimental approach is still necessary for lipophilicity assessment of particular classes of drugs, such as investigated (fluoro)quinolone derivatives. The amphiphilic character of FQs makes assessments of lipophilicity very difficult for theoretical approaches. Most FQs exist in charged form within the physiological pH region. For this reason, their lipophilicity is considerably lower than many neutral and/or basic drugs. This phenomenon was extensively studied by Völgyi and co-workers [10]. 

### 2.2. Analysis of Chromatographic Data and Chromatographically Derived Lipophilicity Indexes

Several reports indicated that RP-TLC can be considered as an efficient tool for prediction of lipophilicity [11,12,13,14,15]. For this reason, two main RP-TLC stationary phases, C_8_ and C_18_-bonded silica gels were investigated during this study. Preliminary chromatographic analyses with buffered mobile phases (phosphoric buffer at pH 7.4.) showed very weak changes of retention compared to unbuffered systems. Similar results have been reported by Hubicka and co-workers for non-buffered TLC lipophilicity determination of FQs [16]. As proposed by Komsta, who examined the impact of several organic modifiers of mobile phase in terms of log*P* predictions [17], methanol was chosen as mobile phase organic component. Various chromatographic lipophilicity indexes, such as *R*_M_^0^, mean values of *R*_M_ (m*R*_M_), hydrophobic constants *b*, and parameter *C*_0_ with likened hydrophobic and lipophilic information, were calculated based on TLC data. Furthermore, principal component analysis (PCA) analysis was implemented by means of a protocol proposed by Sarbu and co-workers [18]. This method allowed obtaining the first principal component, PC_1_, as another lipophilicity index.

The obtained retention factors, *R*_M_ were listed in Table S2. All RP-TLC chromatographic lipophilicity parameters are listed in Table 3, whereas statistical details related to Soczewińki–Wachtmeister’s equation are summarized in Table S3. 

In case of the target compounds, for both newly synthesized hybrids and parent quinolone antibiotics, the Soczewiński–Wachtmeister’s equation showed linear relationships between the *R*_M_ values and the concentration of the organic modifier in the mobile phase. These findings were confirmed with high coefficients of correlation (*R*) and determination (*R*^2^), as well as high values of the *F* statistics. Conversely, small standard deviations or standard estimation errors indicated that all the obtained equations were highly significant. Similar observations for commercially available FQs were reported by Hubicka and co-workers for different RP-TLC chromatographic setups [17]. These results indicated that Soczewiński–Wachtmeister’s equation can be effectively applied for description of chromatographic properties of (fluoro)quinolones derivatives in RP-TLC. 

In order to evaluate the hypothesis that the analytes can be regarded as a group of structurally similar compounds from the chromatographic point of view, linear correlation between slope and intercept of Soczewiński–Wachtmeister’s equation were examined.

*R*_M_^0^(C_18_) = −0.643 (±0.080)*b* + 0.865 (±0.185) (1)

*R* = 0.869, *R*^2^= 0.754, *F* = 64.48, *p* < 0.0001, *s* = 0.273, *N* = 23

*R*_M_^0^(C_8_) = −0.553 (±0.075)*b* + 0.980 (±0.146) (2)

*R* = 0.849, *R*^2^= 0.721, *F* = 54.344, *p* < 0.0001, *s* = 0.227, *n* = 23

Since these parameters were significantly intercorrelated, the investigated structures can be considered as a congeneric class of (fluoro)quinolone derivatives. 

Interestingly, not all TLC chromatographic parameters are highly correlated with each other. The corresponding correlation matrix is presented in Table S4. This observation suggests that several TLC data processing schemes should be considered during the lipophilicity assessment, because each of the parameters may contain diverse information. Taking into account the chromatographic lipophilic parameters, the newly synthesized structures have more hydrophilic character than the parent quinolones. 

Another tested method for lipophilicity assessment was MEKC. The aqueous micelle solution fills the capillary in MEKC and forms the pseudo-stationary phase. Mechanism of migration of analyses in MEKC is determined by lipophilicity of analytes. The more lipophilic the molecules are the stronger they interact with micelles and migrate toward the cathode with lower velocity [3,6]. The advantage of MEKC, compared to reversed-phase liquid chromatography, is elimination of the stationary phase since intercolumn variability can increase measurement errors [6]. Other benefits are shorter time analysis and significantly smaller consumption of organic solvents. For this reason, MEKC was used for lipophilicity assessment of the target compounds [7].

The lipophilicity measurement by MEKC was performed under physiological pH 7.4. The obtained log*k* parameters are summarized in Table 3. Satisfactory correlations between m*R*_M_, PC_1_, and log*k* (*R* < 0.835) were found. The newly synthesized hybrid (fluoro)quinolone-based quaternary ammonium derivatives show less lipophilic character than the parent (fluoro)quinolones. Significantly lower log*k* was noticed in case of pipemidic acid (log*k* = −0.1697) in comparison to other tested (fluoro)quinolone antibiotics. Among the tested (fluoro)quinolone antibiotics, only pipemidic acid belongs to the first generation of quinolone antibiotics which do not incorporate fluorine atom in the structure. Hence, other investigated antibiotics are classified as fluoroquinolones (FQs). Two newly synthesized pipemidic acid derivatives (6 and 10) displayed the most hydrophilic character among all tested structures. On the other hand, the most lipophilic compound in the examined group was enoxacin (log*k* = 0.1564). Among the tested hybrids, sparfloxacin derivative 3 incorporating two fluorine atoms proved the most lipophilic character (log*k* = −0.070). 

### 2.3. Sum of Ranking Differences of Computationally and Chromatographically Derived Lipophilicity Indexes

Although chemometric tools such as HCA and PCA, as well as inspection of a simple correlation matrix, can reveal some similarities and dissimilarities among lipophilicity descriptors [19,20,21]; these methods show considerable limitations. Both CA and PCA do not provide any information regarding the statistical significance of such similarities. Furthermore, these chemometric tools do not indicate or select the most suitable methods for lipophilicity estimation. Such problems can be solved by robust, nonparametric ranking methods, such as the sum of ranking differences (SRD). The usefulness of the SRD analysis for comparison of chromatographically determined lipophilicity parameters and those calculated in silico have been demonstrated by Héberger and Andrić [22,23,24].

The SRD workflow is simple. The data are arranged in a matrix. Methods to be compared are placed in columns, and objects (e.g., chemical compounds) in rows. Then, a benchmark, or reference column, is added. In a case of consensus comparison, it is a vector of arithmetic means. Then, column-wise ranking is performed, and the rank values associated with each particular method are subtracted from the reference ranks. In that way, rank differences are obtained. These differences are then summed up into the SRD values, which are associated with each method. The lower the SRD value, the closer the method to the reference is. In order for results of different SRD analyses to be compared, the normalized SRD value is calculated according to the equation:

(3) $$SRD\left(\%\right)=\frac{SRD}{SRD_{max}}\times 100$$

Such a ranking can be validated in two ways. The first one is comparison of SRDs with theoretical random distribution of SRD values or by a simulation of random numbers. This is so-called comparison with random numbers (SRD-CRRN). The other way is a so-called sevenfold-cross validation. During the cross-validation process, one of seven objects is omitted and the SRD is performed on a truncated data matrix. Such procedure is repeated seven times. The result is seven SRD values associated with each method. Methods are then arranged in ascending order of the median SRD values and the results are depicted in the form of a box and whisker plot. Because the cross-validation extracts the variability of SRDs, it is possible to perform pairwise significance testing of each pair of methods. If no statistically significant difference among the methods is observed, they are grouped together. Otherwise, they are separated into different groups or sections. 

**Figure 2:**
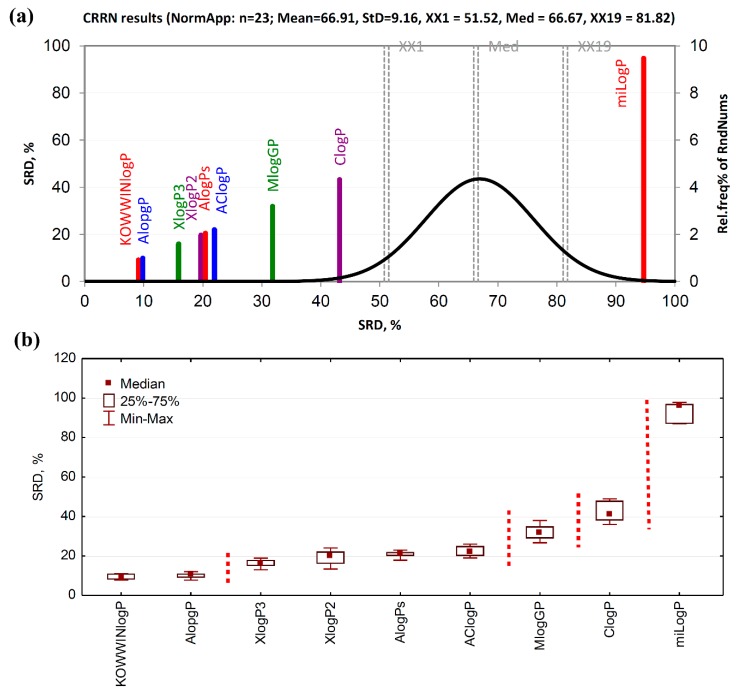
Ranking of computational methods for lipophilicity estimation. (**a**) sum of ranking differences-comparison of ranks by random numbers (SRD-CRRN) of standardized log*P* values; the SRD values are depicted on x and y axes, n = 23, random distribution Mean = 66.91, StD = 9.16, XX1 = 51.52, Med = 66.67, XX2 = 81.82. The smaller the SRD value, the better, i.e., closer to the consensus, the computational method is. (**b**) Box and whisker plot of normalized SRD values obtained by the sevenfold cross-validation. Statistically significantly different methods (*p* = 0.05, tested by both the sign test and the Wilcoxon’s matched pairs test), are separated by dashed lines.

The SRD-CRRN analysis of computational lipophilicity estimation approaches revealed that both, KOWWINlog*P* and Alog*P*, had the smallest SRD values, hence they are closest to the consensus and can be regarded as the best log*P* estimates (Figure 2a). They are closely followed by Xlog*P*3, Xlog*P*2, Alog*P*s, and AClog*P*. Although Mlog*P* and Clog*P* are located a bit lower on the SRD scale, all the mentioned methods are on the left side of the plot, far from the random distribution curve, meaning that all of them are statistically significantly ranked. However, miLog*P* is located at the opposite side of the plot, also significantly distant from the random distribution curve which indicates statistically significant but opposite relation to the consensus and all other methods. Such a different behavior of miLog*P* was already explained in Section 3.1. Sevenfold cross-validation followed by the ordering of the SRD values medians and the pairwise significance testing of each of the studied lipophilicity estimation methods revealed that the first two methods (KOWWINlog*P* and Alog*P*) cannot be statistically significantly distinguished from each other at the significance level of *p* = 0.05, based on both the sign test as well as the Wilcoxon’s matched pairs test. They are placed together in the first section (Figure 2b). This is in accordance with the HCA analysis. Similarly, Xlog*P*3, Xlog*P*2, Alog*P*s, and AClog*P* are grouped together in the second section, while Mlog*P*, Clog*P*, and miLog*P* are all separated from each other. Similar SRD results were observed using interval scaled as well as rank transformed log*P* data (Figures S1 and S2). The ordering of methods and composition of sections may slightly vary depending on data transformation. 

**Figure 3:**
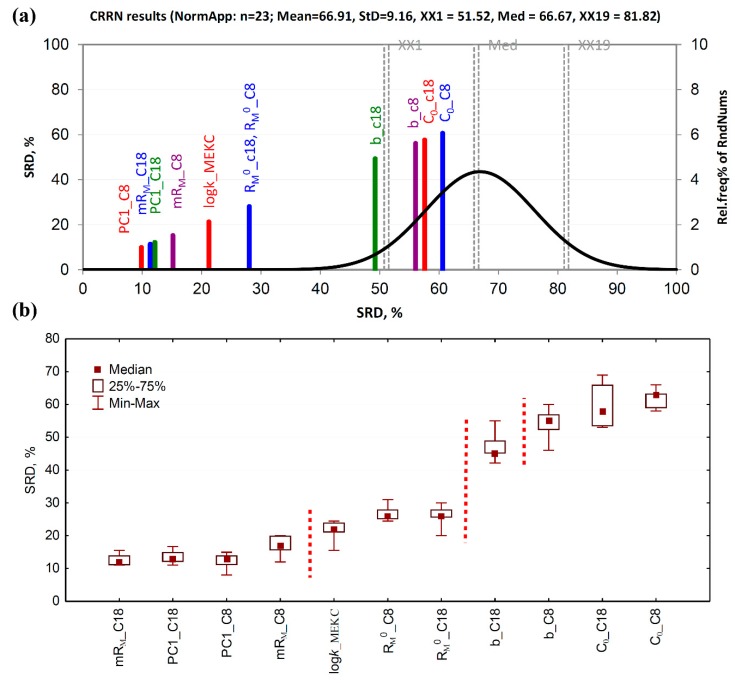
Consensus-based ranking of chromatographic lipophilicity indexes. (**a**) SRD-CRRN of standardized lipophilicity measures; the SRD values are depicted on x and y axes, n = 23, random distribution Mean = 66.91, StD = 9.16, XX1 = 51.52, Med = 66.67, XX19 = 81.82. (**b**) Box and whisker plot of normalized SRD values obtained by the sevenfold cross-validation. Indexes for which the median SRD values are statistically significantly different at the predefined significance level of *p* = 0.05 (tested by both the sign test and the Wilcoxon’s matched pairs test) are separated by dashed lines.

The SRD analysis of chromatographic lipophilicity assessments revealed that PC_1_ scores, as well as m*R*_M_ values, obtained on both stationary phases are among the best lipophilicity measures, closely followed by the extrapolated *R*_M_^0^ values and the log*k* values obtained from the MEKC experiments (Figure 3a). All of them are positioned on the left side of the plot, far from the random distribution curve, which indicates statistically significant ranking of fluoroquinolone derivatives according to their lipophilic character measured by these descriptors. However, the slope obtained on C_8_-modified silica, as well as *C*_0_ values, obtained on both sorbents, falls under the random distribution of SRDs, i.e., these chromatographic descriptors are not capable of distinguishing fluoroquinolone compounds according to the lipophilic character better than a chance. Therefore, such descriptors can be considered as the worst chromatographic lipophilicity measures. Sevenfold cross-validation, followed by nonparametric significance testing of SRD values, by both the Wilcoxon’s matched paired test and sign test, revealed that PC_1_ scores, as well as m*R*_M_ values obtained on both sorbents, belong to the first and the best group of chromatographic descriptors, demonstrating no statistically significant difference among them. They are followed by the second group, consisting of log*k*_MEKC_ and *R*_M_^0^ values obtained on both stationary phases. This confirms that retention of fluoroquinolone derivatives measured by micellar electrokinetic chromatography is equally suitable as extrapolated retention parameters obtained on C_8_- and C_18_-modified silica. However, the ability of interpolation parameters, such as PC_1_ and m*R*_M_, supersedes extrapolated parameters in describing lipophilic character. This is additionally confirmed by the fact that the slope obtained on C_8_-modified silica differed from the slope obtained on C_18_-modified sorbent which is, together with *C*_0_ values obtained on both stationary phases, in the last and the worst group of chromatographic descriptors. Similar findings were obtained using interval scaled and ranked data, with slight deviations in ordering and grouping of lipophilicity indexes depending on the data pretreatment, e.g., PC_1__C_8_ becomes the first ranked in the case of interval scaled and rank transformed data. Also, log*k*_MEKC_ may appear as a separate section (Figures S3 and S4). 

### 2.4. Quantitative Structure Retention/Activity Relationships QSRAR 

Another aspect of the study involved suitability assessment of MEKC derived lipophilicity parameters for prediction of antimicrobial properties of the target (fluoro)quinolone derivatives. MEKC can be considered as a good biomimetic model since Sodium dodecyl sulfate (SDS) micelles have a similar anionic surfactant structure to natural phospholipids [25]. Generally, lipophilicity is a well-recognized physicochemical parameter, which influences antibacterial properties of different classes of antibiotics, including FQs [10,26]. Bilinear structure–activity relationships between distribution coefficient (log*D* at pH 7.4) and antibacterial properties were reported for 4′N-alkylciprofloxacin analogs [26]. Another study showed relationship between minimum inhibitory concentration (MIC) of two gram-positive bacteria species (*Streptococcus pneumoniae* and methicillin-susceptible *Staphylococcus aureus*) and the true partition coefficient of the selected FQs [10]. The above method is limited by complicated protocol for true partition coefficient assessment, as it requires determination of protonation microconstants and log*D* at the isoelectric point. The proposed MEKC method has significant advantages because it is simple, highly efficient, offers short time of analysis, and requires small amounts of samples and chemical reagents. Based on the microbiological data previously reported [7], an acceptable correlation was found between the log*k* and log*MIC* for three bacterial species as listed below.

log*MIC*_(*Bacillus subtilis*)_ = − 4.286(±0.632)log*k* − 0.444(±0.170) (4)

*R* = 0.823, *R*^2^ = 0.677, *Q*^2^ = 0.643, *F* = 46.385, *p* < 0.001, *s* = 0.650, *n* = 23

log*MIC*_(*Escherichia coli*)_ = − 4.900(±0.553)log*k* − 0.745(±0.150) (5)

*R* = 0.888, *R*^2^ = 0.789, *Q*^2^ = 0.750, *F* = 78.405, *p* < 0.001, *s* = 0.571, *n* = 23

log*MIC*_(*Proteus vulgaris*)_ = − 1.346(±0.632)log*k* − 4.596(±0.794) (6)

*R* = 0.784, *R*^2^ = 0.615, *Q*^2^ = 0.541, *F* = 33.5115, *p* < 0.001, *s* = 0.650, *n* = 23

**Figure 4:**
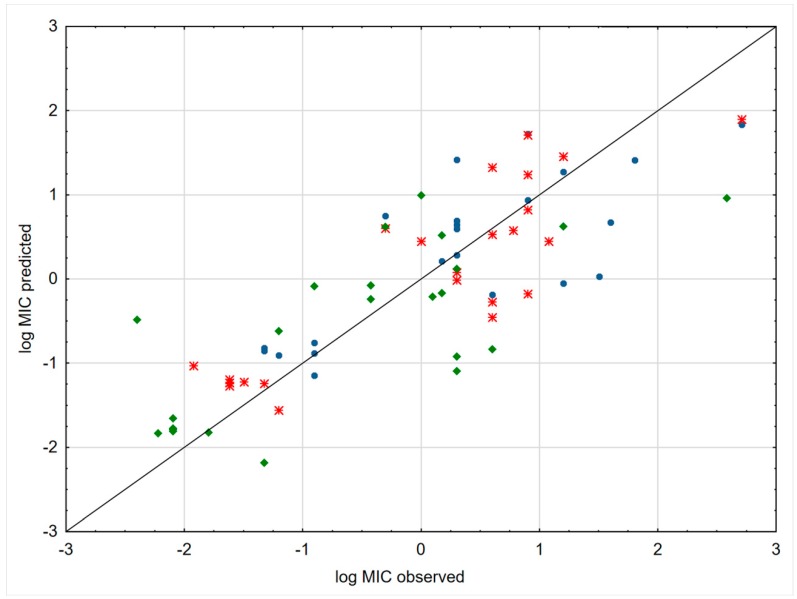
The performance comparison of the obtained QSAR models for each of the bacterial strains. Scatter plots comparing observed log MIC for *Proteus vulgaris* (green diamonds), *Bacillus subtilis* (blue circle), and *Escherichia coli* (red points) value with predicted value.

The obtained models met the Tropsha et al. criteria (R^2^ > 0.6 and Q^2^ > 0.5) [27]. Figure 4 presented plots of predicted and experimental values of MIC. As might be expected, the obtained models do not explain completely antibacterial properties of (fluoro)quinolone derivatives. The retention data give information only about lipophilicity. MEKC experiment can mimic drug–t membranes interactions. FQs have a typical drug–receptor mechanism of action, i.e., they inhibit the action of type II topoisomerases, DNA gyrase, and DNA topoisomerase IV [28]. The steric and electrostatic interactions between FQs and enzymes strongly affect antibacterial properties. Nevertheless, the obtained results indicated that lipophilicity is one of the vital parameters which considerably influences antibacterial properties. The plot showing the relation between the observed and predicted values of log*MIC* is presented in Figure 4. Our QRAR models confirm the observation reported by Völgyi [10] that lipophilicity increase within a series of (fluoro)quinolone derivatives can be associated with an improved antibacterial profile. These results clearly suggest that there is a correlation between lipophilicity and antibacterial properties of FQs. Presumably more lipophilic structures diffuse more easily into bacterial cells [29]. 

## 3. Materials and Methods 

### 3.1. Reagents

Sodium dodecyl sulfate (SDS), 4-(2-hydroxyethyl)piperazine-1-ethanesulfonic acid (HEPES), and tris(hydroxymethyl)aminomethane (TRIS) were supplied by Sigma-Aldrich (Steinheim, Germany). Dimethyl sulfoxide (DMSO) was purchased from POCH (Gliwice, Poland). Methanol and acetone analytical grade for liquid chromatography was purchased from Sigma-Aldrich (Steinheim, Germany). Ultrapure water purified by Millipore Direct-Q 3 UV Water Purification System (Millipore Corporation, Bedford, MA, USA) was used for mobile phase preparation.

### 3.2. Analytes

The analytical standards of pipemidic acid, enoxacin, and gatifloxacin were provided by Alfa Aesar (Haverhill, MA, USA); norfloxacin, lomefloxacin, ciprofloxacin, sparfloxacin, and moxifloxacin were purchased from Sigma-Aldrich (Steinheim, Germany). Synthesis and purification of the hybrid quinolone-based quaternary ammonium derivatives were described previously [7]. The structures of the investigated hydrides are presented in Table S1. All target compounds were dissolved in DMSO to obtain a concentration of 1 mg mL^−1^ and stored at 2–8 °C prior to analyses. 

### 3.3. TLC Analysis 

The ready-to-use TLC glass plates covered by octadecyl(C_18_)- and octyl(C_8_)-modified TLC silica layers were purchased from Merck (Darmstadt, Germany). Stock solutions (5 µL) of the analytes were spotted manually on the plates with the use of a micropipette from Brand (Wertheim, Germany). The mobile phases were prepared by mixing appropriate volumes of pure organic solvents and water (in step gradient by 10% (v/v)), in the following proportions: (a) methanol–water ranging from 30 to 70% (v/v), in the case of C_8_-bonded silica gel stationary phase; and (b) methanol–water ranging from 40 to 70% (v/v), in the case of C_18_-bonded silica gel stationary phase.

Before analysis, the chromatographic chamber (Twin Trough Chambers from CAMAG Philadelphia, USA) was saturated for 20 min. All TLC experiments were performed at room temperature (22 ± 2 °C). Chromatograms were developed using the ascending technique to a distance of 8 cm. The investigated substances were visualized under UV light at λ = 366 nm by CAMAG UV Lamp 4 and the Viewing Box 4 (Philadelphia, USA). The target compounds were visible as blue spots. The obtained retardation factors (*R*_F_) were collected in the Supplementary data sheets. The retention parameter, *R*_M,_ was calculated for each compound according to the Bate-Smith and Westall formula:

(7) $$R_{M}=log\left(\frac{1}{R_{F}}-1\right)$$

and listed in Table S2. Next, the Soczewiński–Wachtmeister’s [30] equation:

*R*_M_ = *R*_M_^0^ + *b*C (8)

was used in order to determine the basic lipophilic RP-TLC parameter *R*_M_^0^ as well as commonly used hydrophobic constants *m*. Soczewiński–Wachtmeister’s equation presented a linear relationship between concentration of the organic solvent in mobile phase (C) and *R*_M_ value. Moreover, mean value of R_M_ was used for lipophilicity assessments. 

Furthermore, *C*_0_ RP-TLC lipophilicity parameter, introduced by Bieganowska [31], has been calculated according to the formula:

(9) $$C_{0}=-\frac{{R_{M}}^{0}}{b}.$$

The *C*_0_ parameter corresponds to the parameter φ_0_, previously proposed for the HPLC. This parameter relates to the concentration of the organic component in the mobile phase for which the distribution of the analyzed substance between the mobile and stationary phase is equal (1:1) [31].

Also, the principal component analysis (PCA) was applied in order to extract information regarding the lipophilicity of solutes and formulate them as the first principal component (PC_1_). Calculation data matrixes included *R*_M_ values of solutes for each modifier concentration in the mobile phase. The obtained TLC lipophilicity constants *R*_M_^0^_,_ *m*, m*R*_M_, *C*_0,_ and PC_1_ are listed in Table 2. 

### 3.4. MEKC Analysis

All MEKC experiments were carried out with a P/ACE MDQ plus system (Sciex, Framingham, MA, USA) equipped with photodiode array detector (PDA) and controlled by 32 Karat software (version 10.2). The uncoated fused silica capillaries (50 µm i.d., Polymicro Technologies, West Yorkshire, UK) of 60 cm total length and 50.2 cm effective length were used. Prior to analysis, the capillary was rinsed with 0.1 M NaOH for 30 min, ultrapure water for 10 min, and background electrolyte (BGE) for 30 min. After each analysis, the capillary was conditioned with BGE for 2 min. The applied pressure for all rinsing operations was 345 kPa. The analytes were dissolved in BGE at concentration of 100 µg/mL with addition of quinine (micelles marker). As electroosmotic flow (EOF) marker, DMSO was used. The samples were hydrodynamically injected at 35 kPa for 5 s. The separations were performed by applying voltage of 20 kV with positive polarity and constant temperatures at 25 ± 0.1 °C. The BGE consisted of aqueous solution of 50 mM SDS and 120 mM HEPES /100 mM TRIS buffer of pH 7.4. The BGE was prepared each working day by dilution of stock solution. Detection was carried out at 200 and 280 nm with 8 Hz probing frequency. The logarithm of retention factor log*k* was calculated according to the equation introduced by Terabe and co-workers:

(10) $$logk_{MEKC}=log\left(\frac{t_{R}-t_{EOF}}{t_{EOF}\left(1-t_{R}/t_{MC}\right)}\right)$$

The established log*k* parameters are summarized in Table 2. 

### 3.5. Computational Estimation of logP Values

The log*P* values were calculated using several software platforms. Six different log*P* values (AlogPs, AClogP, AlogP, MLOGP, XlogP2, and XlogP3) and their average (Avglog*P*) were estimated using the Virtual Computational Chemistry Laboratory (VCCLAB, http://www.vcclab.org/), accessed on 11 September 2019. ClogP parameter was obtained with ChemDraw (Waltham, MA, USA), while miLogP values were calculated using Molinspiration algorithm (http://www.molinspiration.com/), accessed on 11 September 2019. The KOWWINlogPs were calculated using the KOWWIN software v. 1.68 (EPI Suite package v.4.1, U.S. EPA).

### 3.6. Data Analysis

The chromatographic parameters (*R*_M_, mean value of *R*_M,_ *C*_0_, and log*k*) were calculated from retention data using Microsoft Excel 2007 (Microsoft Redmond, WA, USA). All presented linear regressions were calculated using STATISTICA 9.1 (Stat-Soft, Tulsa, OK, USA). The coefficients of correlation (*R*) and determination (*R*^2^), *F*-test value, standard deviation (σ), and standard estimation error (*s*) were used as the basis for testing the quality of obtained linear regression models. All presented linearities were performed at a significance level less than 0.05 whereas the critical F-values for performed F-tests was < 2. 

Cluster analysis (HCA) was carried out using STATISTICA 9.1 based on the calculated lipophilicity indexes. Before performing the HCA, lipophilicity data were standardized, i.e., the average was subtracted from each value and the difference was divided by the standard deviation. In that way, differently scaled variables were placed on the same, so-called unit standard deviation scale. Euclidean distance was used to measure distance between objects while Ward’s amalgamation method was applied to build up clusters. 

The sum of ranking differences (SRD) was performed using Microsoft Excel visual basic macros freely available from http://aki.ttk.mta.hu/srd/. Before the analysis, the data were organized in a matrix with compounds arranged in rows and lipophilicity measures in columns. The SRD analysis was performed separately on computationally estimated log*P*s and chromatographic lipophilicity measures. Since the SRD analysis requires a benchmark vector for methods to be compared with, the average of all methods (consensus vector) was selected as the benchmark (reference). Comparison of methods to consensus has two major advantages. First, the average has the maximum likelihood of being the true value. Second, all the errors associated with each method, either systematic or random ones, cancel each other out by averaging. In order for consensus to be calculated all the data must be expressed on the same scale. As the result of SRD, analysis may slightly vary depending on the data transformation; three data pretreatment methods were tested: Standardization (as previously described), interval scaling between 0 and 1 (in ascending order), and rank transformation (taking average rank values for ties). STATISTICA 9.1 was used for statistical significance testing of the SRD values obtained by the sevenfold cross-validation and plotting of box and whisker diagrams. 

## 4. Conclusions

Lipophilicity of a series of fluoroquinolone-*Safirinium* dye hybrids, synthesized as new dual-acting hydrophilic antibacterial agents, have been assessed based on chromatographic and computational approaches. Vast differences between calculated log*P* parameters indicated that experimental procedures are still required for lipophilicity determination of this chemical group. Soczewiński–Wachtmeister’s equation well presented the chromatographic behavior of the studied compounds in RP-TLC. The SRD analysis of chromatographic lipophilicity measures revealed that PC_1_ scores as well as m*R*_M_ values, obtained on both stationary phases, are among the best lipophilicity measures, closely followed by the extrapolated *R*_M_^0^ values and the log*k* values obtained from the MEKC chromatographic experiments. Furthermore, acceptable correlation was found between the log*k* parameter and log*MIC* for three bacterial strains, i.e., *Bacillus subtilis*, *Escherichia coli*, and *Proteus vulgaris*. These results suggested that MEKC can serve as a screening tool for the prediction of antimicrobial activity of fluoroquinolone derivatives.

## Figures and Tables

**Table 1:** Calculated log*P* values for investigated compounds.

Compound No.	Clog*P*	miLog*P*	KOWWINlog*P*	Alog*P*s	AClog*P*	Alopg*P*	Mlog*P*	Xlog*P*2	Xlog*P*3	Avglog*P*
1	−3.10	−5.08	1.34	2.35	1.92	0.60	−0.04	2.27	3.57	1.78
2	−2.55	−4.96	2.14	2.83	2.29	1.10	−0.18	2.77	4.16	2.16
3	−2.89	−3.92	1.76	3.20	2.02	0.95	0.43	2.66	4.04	2.22
4	−3.51	−5.21	0.83	2.29	2.14	0.02	−0.09	1.63	2.01	1.33
5	−2.43	−4.92	1.96	2.85	2.26	1.18	0.26	2.89	4.10	2.26
6	−5.06	−4.67	−0.06	1.48	1.14	−0.33	0.05	1.27	2.42	1.01
7	−4.10	−4.97	1.44	2.13	1.80	0.52	0.67	2.48	3.17	1.79
8	−3.05	−5.09	1.65	2.36	2.11	0.74	0.16	2.39	3.76	1.92
9	−2.89	−5.07	1.14	2.85	2.29	0.47	0.01	2.13	3.29	1.83
10	−5.52	−4.86	−0.87	1.68	1.17	−1.04	−0.32	0.51	0.94	0.51
11	−4.56	−5.12	0.62	2.19	1.82	−0.19	0.40	1.71	2.89	1.47
12	−3.56	−5.21	0.52	2.33	1.94	−0.12	−0.29	1.51	2.06	1.24
13	−3.01	−5.11	1.33	2.75	2.32	0.38	−0.42	2.00	3.32	1.73
14	−3.35	−4.28	0.95	3.05	2.05	0.24	0.19	1.89	3.20	1.77
15	−4.07	−4.80	2.26	2.80	2.56	1.30	0.20	3.80	4.54	2.41
16	−4.03	−2.83	0.80	0.82	0.58	0.07	−1.86	2.15	0.61	0.39
17	−2.57	−3.11	0.16	0.99	0.28	−0.06	−0.89	1.96	−0.80	0.08
18	−3.25	−3.56	−0.46	0.57	−0.06	−0.64	−2.25	1.34	−1.03	−0.35
19	−5.21	−2.56	−1.86	−0.84	−0.84	−1.56	−2.63	0.34	−2.15	−1.28
20	−3.04	−1.53	−0.04	0.79	0.04	−0.28	−1.68	1.73	0.11	0.12
21	−2.70	−3.22	0.34	0.88	0.31	−0.14	−2.31	1.84	−0.24	0.06
22	−3.19	−3.57	−0.15	0.20	0.13	−0.50	−2.01	1.46	−1.08	−0.30
23	−4.24	−3.25	−0.36	0.21	−0.18	−0.71	−1.63	1.55	−0.20	−0.16

**Table 2:** Information about algorithms and suppliers of investigated software.

No.	logP Scale	Algorithms	Supplier
1	ClogP	fragment contribution	www.biobyte.com (ChemDraw)
2	milogP	fragment contribution	www.molinspiration.com
3	KOWWINlogP	hybrid algorithm (atom-based approach and fragmental contribution)	www.epa.gov
4	AlogPs	properties dependent methods (topological descriptors)	www.vcclab.org
5	AClogP	atom-based method	www.acdlabs.com
6	AlopgP	fragment contribution	www.vcclab.org
7	MlogP	properties dependent methods (topological descriptors)	http://www.talete.mi.it/
8	XlogP	atom-based method	http://www.compchemcons.com
9	XlogP3	atom-based method	http://www.compchemcons.com

**Table 3:** Chromatographically determined lipophilicity parameters.

Compound No.	RP-TLC Stationary Phase: Silica Gel C_18_					RP-TLC Stationary Phase: Silica Gel C_8_					MEKC	
	*R*_M_^0^	*b*	*C*_0_	m*R*_M_	PC_1_ *	*R*_M_^0^	*b*	*C*_0_	m*R*_M_	PC_1_ **	log*k*	σ log*k*
1	1.65	−1.36	1.21	0.96	0.72	1.99	−1.92	1.04	1.03	−2.23	−0.104	0.0057
2	2.78	−3.31	0.84	0.95	0.81	2.31	−2.61	0.89	1.01	−2.22	−0.125	0.0036
3	2.75	−3.13	0.88	1.02	0.34	2.74	−3.17	0.86	1.15	−3.48	−0.070	0.0041
4	1.74	−1.85	0.94	0.72	2.36	1.79	−1.90	0.94	0.84	−0.58	−0.321	0.0117
5	2.29	−2.45	0.93	0.94	0.88	1.90	−1.83	1.04	0.98	−1.69	−0.265	0.0020
6	1.78	−1.45	1.23	0.98	0.53	1.32	−0.56	2.35	1.04	−1.73	−0.443	0.0003
7	1.87	−1.60	1.17	0.99	0.48	1.76	−1.43	1.23	1.05	−2.01	−0.239	0.0009
8	1.83	−1.71	1.07	0.89	1.18	1.71	−1.56	1.09	0.93	−1.26	−0.153	0.0007
9	1.86	−1.91	0.98	0.81	1.75	1.62	−1.66	0.97	0.79	−0.11	−0.479	0.0039
10	1.27	−1.04	1.22	0.70	2.53	1.16	−0.91	1.28	0.71	0.79	−0.573	0.0021
11	1.72	−1.59	1.08	0.84	1.51	1.59	−1.50	1.06	0.84	−0.54	−0.398	0.0027
12	1.75	−1.75	1.00	0.78	1.97	1.63	−1.63	1.00	0.82	−0.38	−0.272	0.0012
13	2.18	−2.47	0.88	0.82	1.74	1.82	−1.97	0.92	0.83	−0.64	−0.408	0.0175
14	2.45	−2.84	0.86	0.88	1.32	2.29	−2.66	0.86	0.96	−1.79	−0.261	0.0070
15	3.56	−4.21	0.84	1.24	−1.12	2.63	−2.88	0.91	1.19	−3.52	−0.250	0.0036
16	2.62	−2.43	1.08	1.30	−1.59	1.99	−1.49	1.33	1.24	−3.61	0.108	0.0001
17	2.33	−2.03	1.15	1.22	−1.08	2.06	−1.54	1.34	1.29	−4.09	0.077	0.0003
18	2.54	−2.06	1.24	1.40	−2.34	2.43	−1.90	1.27	1.47	−5.56	0.103	0.0049
19	2.39	−1.78	1.35	1.24	−1.24	1.87	−1.13	1.65	1.30	−3.91	−0.170	0.0044
20	2.85	−2.88	0.99	1.28	−1.40	2.27	−2.13	1.07	1.21	−3.81	0.116	0.0005
21	2.69	−2.55	1.06	1.31	−1.63	2.68	−2.83	0.95	1.26	−4.29	0.101	0.0012
22	2.79	−2.15	1.30	1.62	−3.88	2.04	−1.27	1.61	1.41	−5.04	0.104	0.0011
23	2.70	−1.98	1.37	1.62	−3.86	2.45	−2.00	1.23	1.45	−5.38	0.156	0.0070

* contains 86.92% of information included in the retention data. ** contains 83.57% of information included in the retention data.

## Supplementary Materials

Supplementary materials can be found at https://www.mdpi.com/1422-0067/20/21/5288/s1. Table S1. Chemical names and structural formulas of the studied compounds. Table S2. Retention parameters *R_M_* obtained in RP-TLC systems. Table S3. Statistical parameters of fitted Soczewiński–Wachtmeister’s equation for each of the studied compounds. Table S4. Correlation matrix of TLC chromatographic parameters. Figure S1. Ranking of computational methods for lipophilicity estimation. Figure S2. Ranking of chromatographic lipophilicity indexes. Figure S3. Ranking of computational methods for lipophilicity estimation. Figure S4. Ranking of chromatographic lipophilicity indexes.
