# Supplementary material for: Lipophilicity Determination of Quaternary (Fluoro)Quinolones by Chromatographic and Theoretical Approaches
Source: Int J Mol Sci. 2019 Oct 24;20(21):5288. doi: 10.3390/ijms20215288 (PMC6862250; doi:10.3390/ijms20215288)
Supplement: Supplementary file 1 [file ijms-20-05288-s001.pdf]

Table S1. Chemical names and structural formulas of the studied compounds.

Compound number	Chemical name	Chemical structure
**1**	4-carboxy-4'-(3-carboxy-1-ethyl-6-fluoro-4-oxo-1,4-dihydroquinolin-7-yl)-1*H*-spiro[[1,2,4]triazolo[4,3-*a*]quinoline-2,1'-piperazin]-1'-ium chloride	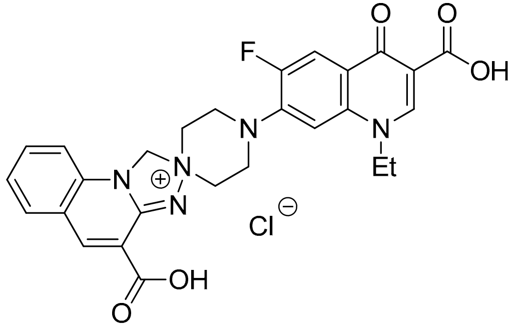
**2**	4-carboxy-4'-(3-carboxy-1-cyclopropyl-6-fluoro-8-methoxy-4-oxo-1,4-dihydroquinolin-7-yl)-2'-methyl-1*H*-spiro[[1,2,4]triazolo[4,3-*a*]quinoline-2,1'-piperazin]-1'-ium chloride	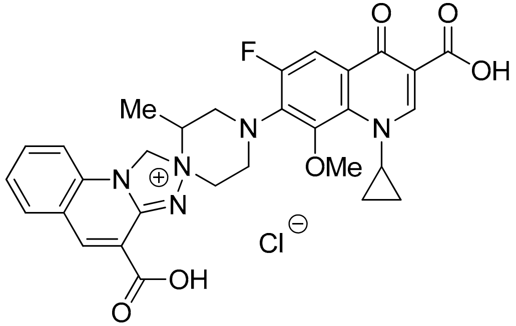
**3**	4'-(5-amino-3-carboxy-1-cyclopropyl-6,8-difluoro-4-oxo-1,4-dihydroquinolin-7-yl)-4-carboxy-2',6'-dimethyl-1*H*-spiro[[1,2,4]triazolo[4,3-*a*]quinoline-2,1'-piperazin]-1'-ium chloride	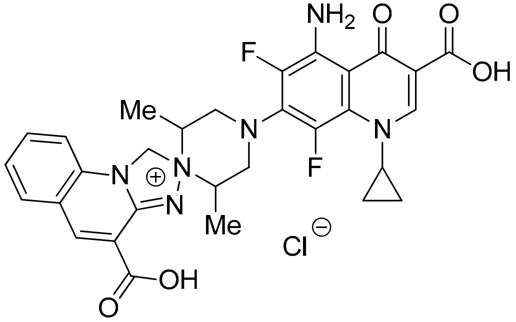

Compound number	Chemical name	Chemical structure
**4**	8-carboxy-4'-(3-carboxy-1-cyclopropyl-6-fluoro-4-oxo-1,4-dihydroquinolin-7-yl)-5,7-dimethyl-3*H*-spiro[[1,2,4]triazolo[4,3-*a*]pyridine-2,1'-piperazin]-1'-ium chloride	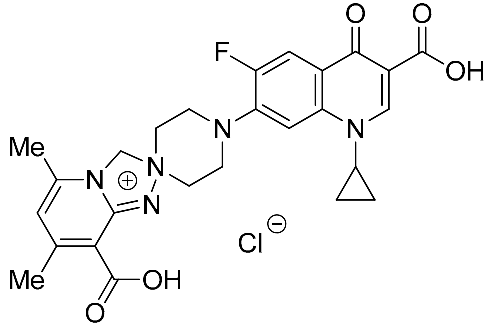
**5**	4-carboxy-4'-(3-carboxy-1-ethyl-6,8-difluoro-4-oxo-1,4-dihydroquinolin-7-yl)-2'-methyl-1*H*-spiro[[1,2,4]triazolo[4,3-*a*]quinoline-2,1'-piperazin]-1'-ium chloride	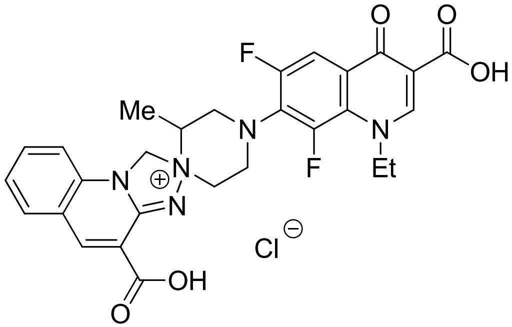
**6**	4-carboxy-4'-(6-carboxy-8-ethyl-5-oxo-5,8-dihydropyrido[2,3-*d*]pyrimidin-2-yl)-1*H*-spiro[[1,2,4]triazolo[4,3-*a*]quinoline-2,1'-piperazin]-1'-ium chloride	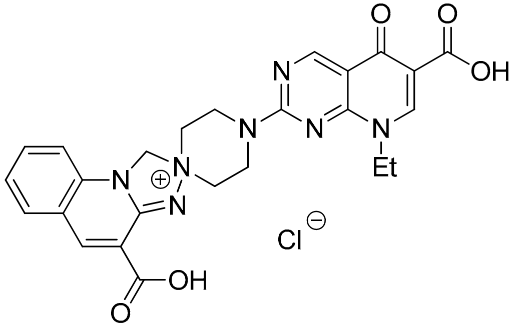
**7**	4-carboxy-4'-(6-carboxy-8-ethyl-3-fluoro-5-oxo-5,8-dihydro-1,8-naphthyridin-2-yl)-1*H*-spiro[[1,2,4]triazolo[4,3-*a*]quinoline-2,1'-piperazin]-1'-ium chloride	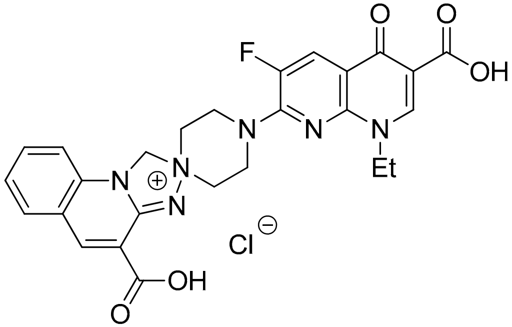

Compound number	Chemical name	Chemical structure
**8**	4-carboxy-4'-(3-carboxy-1-cyclopropyl-6-fluoro-4-oxo-1,4-dihydroquinolin-7-yl)-1*H*-spiro[[1,2,4]triazolo[4,3-*a*]quinoline-2,1'-piperazin]-1'-ium chloride	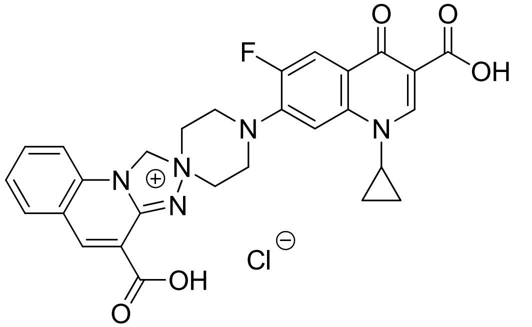
**9**	carboxy-4'-(3-carboxy-1-ethyl-6,8-difluoro-4-oxo-1,4-dihydroquinolin-7-yl)-2',5,7-trimethyl-3*H*-spiro[[1,2,4]triazolo[4,3-*a*]pyridine-2,1'-piperazin]-1'-ium chloride	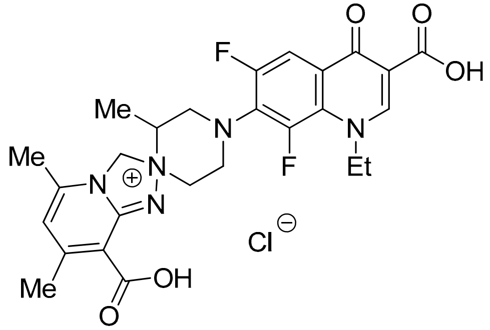
**10**	8-carboxy-4'-(6-carboxy-8-ethyl-5-oxo-5,8-dihydropyrido[2,3-d]pyrimidin-2-yl)-5,7-dimethyl-3*H*-spiro[[1,2,4]triazolo[4,3-*a*]pyridine-2,1'-piperazin]-1'-ium chloride	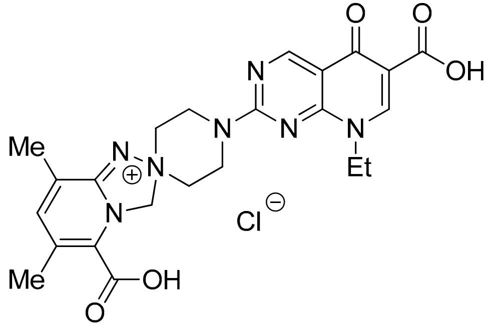
**11**	8-carboxy-4'-(6-carboxy-8-ethyl-3-fluoro-5-oxo-5,8-dihydro-1,8-naphthyridin-2-yl)-5,7-dimethyl-3*H*-spiro[[1,2,4]triazolo[4,3-*a*]pyridine-2,1'-piperazin]-1'-ium chloride	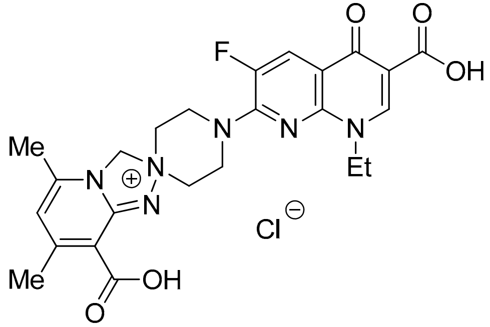

Compound number	Chemical name	Chemical structure
**12**	8-carboxy-4'-(3-carboxy-1-ethyl-6-fluoro-4-oxo-1,4-dihydroquinolin-7-yl)-5,7-dimethyl-3*H*-spiro[[1,2,4]triazolo[4,3-*a*]pyridine-2,1'-piperazin]-1'-ium chloride	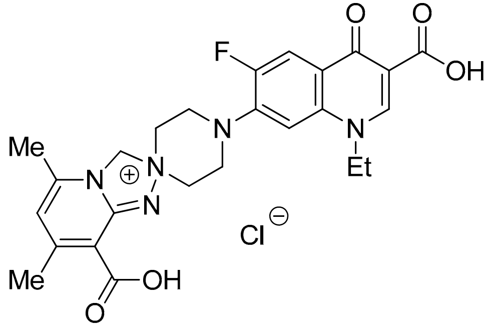
**13**	8-carboxy-4'-(3-carboxy-1-cyclopropyl-6-fluoro-8-methoxy-4-oxo-1,4-dihydroquinolin-7-yl)-2',5,7-trimethyl-3*H*-spiro[[1,2,4]triazolo[4,3-*a*]pyridine-2,1'-piperazin]-1'-ium chloride	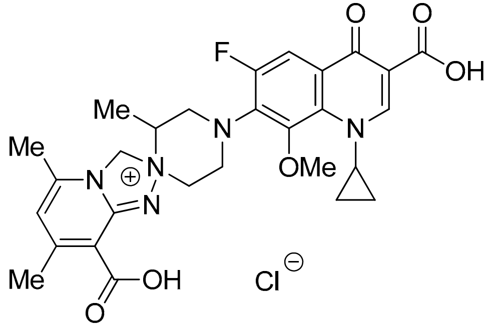
**14**	4'-(5-amino-3-carboxy-1-cyclopropyl-6,8-difluoro-4-oxo-1,4-dihydroquinolin-7-yl)-8-carboxy-2',5,6',7-tetramethyl-3*H*-spiro[[1,2,4]triazolo[4,3-*a*]pyridine-2,1'-piperazin]-1'-ium chloride	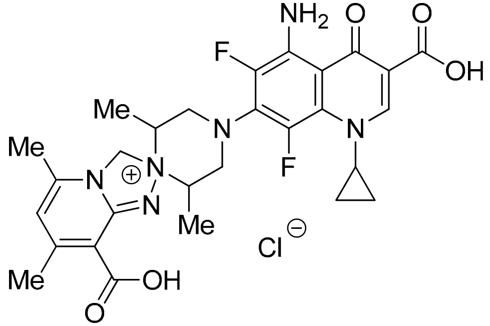
**15**	4-carboxy-6'-(3-carboxy-1-cyclopropyl-6-fluoro-8-methoxy-4-oxo-1,4-dihydroquinolin-7-yl)-2',3',4',4a',5',6',7',7a'-octahydro-1*H*-spiro[[1,2,4]triazolo[4,3-*a*]quinoline-2,1'-pyrrolo[3,4-*b*]pyridin]-1'-ium chloride	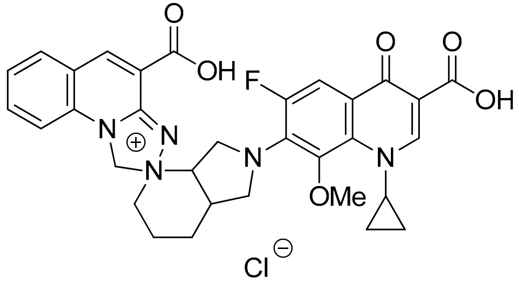

Compound number	Chemical name	Chemical structure
**16**	4-cyclopropyl-7-fluoro-6-((4a*S*,7a*S*)-hexahydro-1*H*-pyrrolo[3,4-*b*]pyridin-6(2*H*)-yl)-5-methoxy-1-oxo-1,4-dihydronaphthalene-2-carboxylic acid (moxifloxacin)	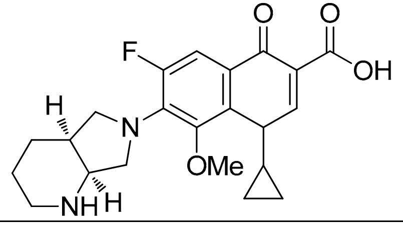
**17**	1-ethyl-6,8-difluoro-7-(3-methylpiperazin-1-yl)-4-oxo-1,4-dihydroquinoline-3-carboxylic acid (lomefloxacin)	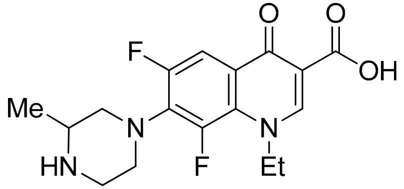
**18**	1-ethyl-6-fluoro-4-oxo-7-(piperazin-1-yl)-1,4-dihydroquinoline-3-carboxylic acid (norfloxacin)	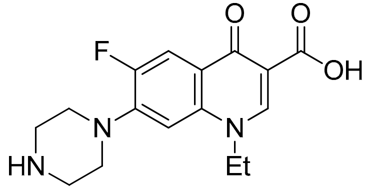
**19**	8-ethyl-5-oxo-2-(piperazin-1-yl)-5,8-dihydropyrido[2,3-*d*]pyrimidine-6-carboxylic acid (pipemidic acid)	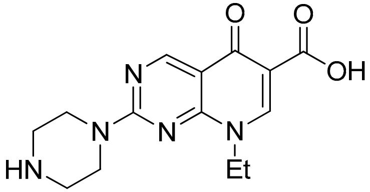
**20**	5-amino-1-cyclopropyl-7-((3*R*,5*S*)-3,5-dimethylpiperazin-1-yl)-6,8-difluoro-4-oxo-1,4-dihydroquinoline-3-carboxylic acid (sparfloxacin)	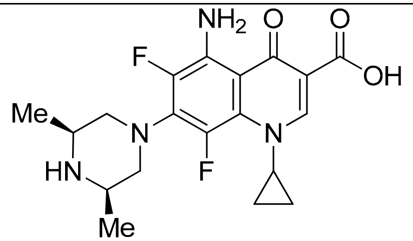
**21**	1-cyclopropyl-6-fluoro-8-methoxy-7-(3-methylpiperazin-1-yl)-4-oxo-1,4-dihydroquinoline-3-carboxylic acid (gatifloxacin)	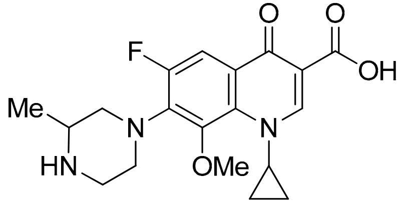

**Compound number**	**Chemical name**	**Chemical structure**
**22**	1-cyclopropyl-6-fluoro-4-oxo-7-(piperazin-1-yl)-1,4-dihydroquinoline-3-carboxylic acid (ciprofloxacin)	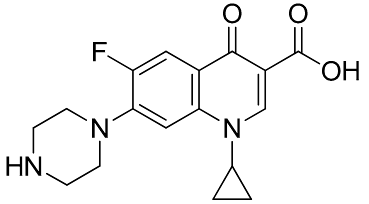
**23**	1-ethyl-6-fluoro-4-oxo-7-(piperazin-1-yl)-1,4-dihydro-1,8-naphthyridine-3-carboxylic acid (enoxacin)	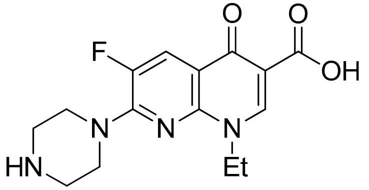

Table S2. Retention parameters R_M obtained in RP-TLC systems

**stationary phase: silica gel C_8 mobile phase: methanol-water**																							
**Percentage of the organics**	**Compound number**																						
	**1**	**2**	**3**	**4**	**5**	**6**	**7**	**8**	**9**	**10**	**11**	**12**	**13**	**14**	**15**	**16**	**17**	**18**	**19**	**20**	21	22	23
30%	1.40	1.59	1.89	1.28	1.41	1.18	1.41	1.28	1.18	0.95	1.17	1.17	1.27	1.59	1.89	1.59	1.59	1.90	1.59	1.59	1.89	1.59	1.89
40%	1.26	1.26	1.39	1.00	1.16	1.08	1.16	1.07	0.94	0.78	1.00	1.00	1.00	1.16	1.39	1.26	1.39	1.57	1.32	1.39	1.39	1.56	1.57
50%	0.99	0.92	1.07	0.76	0.87	1.03	0.92	0.86	0.71	0.63	0.80	0.75	0.80	0.91	1.13	1.37	1.37	1.55	1.37	1.37	1.37	1.55	1.56
60%	0.83	0.73	0.83	0.62	0.79	0.97	0.91	0.80	0.59	0.59	0.66	0.62	0.62	0.65	0.79	1.04	1.13	1.24	1.13	0.97	0.97	1.24	1.13
70%	0.66	0.55	0.59	0.52	0.68	0.95	0.82	0.63	0.52	0.59	0.59	0.55	0.48	0.51	0.76	0.95	0.95	1.11	1.11	0.73	0.68	1.11	1.11
**stationary phase: silica gel C_{18} mobile phase: methanol-water**																							
Percentage of the organics	Compound number																						
	1	2	3	4	5	6	7	8	9	10	11	12	13	14	15	16	17	18	19	20	21	22	23
40%	1.37	1.56	1.56	1.05	1.37	1.24	1.24	1.13	1.13	0.85	1.13	1.04	1.24	1.36	1.85	1.54	1.54	1.85	1.52	1.53	1.53	1.84	1.83
50%	0.94	1.01	1.10	0.76	1.01	1.01	1.11	1.02	0.88	0.77	0.88	0.88	0.88	0.95	1.53	1.52	1.20	1.34	1.35	1.51	1.51	1.82	1.81
60%	0.78	0.68	0.83	0.60	0.73	0.89	0.83	0.78	0.68	0.60	0.73	0.68	0.68	0.73	0.95	1.21	1.21	1.34	1.06	1.34	1.34	1.52	1.52
70%	0.74	0.56	0.60	0.49	0.65	0.79	0.79	0.65	0.56	0.56	0.65	0.52	0.48	0.49	0.64	0.91	0.91	1.07	1.02	0.72	0.84	1.30	1.30

Table S3. Statistical parameters of fitted Soczewiński–Wachtmeister's equation for each of the studied compounds.

**Stationary phase: C_8 modified silica<br>Mobile phase consisted of methanol and water**									
**Compound number**	R_M^0	σR_M^0	b	σb	R	R^2	F	s	p
1	1.987	0.058	-1.915	0.112	0.995	0.990	294.865	0.035	0.000
2	2.313	0.114	-2.606	0.220	0.989	0.979	140.297	0.070	0.001
3	2.740	0.162	-3.173	0.311	0.986	0.972	103.906	0.098	0.002
4	1.786	0.115	-1.900	0.221	0.980	0.961	74.044	0.070	0.003
5	1.897	0.133	-1.831	0.256	0.972	0.945	51.140	0.081	0.006
6	1.322	0.037	-0.562	0.072	0.977	0.954	61.603	0.023	0.004
7	1.759	0.153	-1.425	0.294	0.942	0.887	23.577	0.093	0.017
8	1.710	0.082	-1.563	0.158	0.985	0.970	97.650	0.050	0.002
9	1.617	0.114	-1.660	0.219	0.975	0.950	57.513	0.069	0.005
10	1.165	0.119	-0.911	0.230	0.916	0.839	15.690	0.073	0.029
11	1.592	0.077	-1.495	0.149	0.986	0.971	101.351	0.047	0.002
12	1.631	0.095	-1.627	0.184	0.981	0.963	78.499	0.058	0.003
13	1.821	0.073	-1.974	0.141	0.992	0.985	196.583	0.045	0.001
14	2.291	0.149	-2.656	0.288	0.983	0.966	85.341	0.091	0.003
15	2.631	0.233	-2.879	0.448	0.966	0.932	41.319	0.142	0.008
16	1.990	0.185	-1.494	0.356	0.924	0.854	17.578	0.113	0.025
17	2.056	0.093	-1.539	0.179	0.980	0.961	73.912	0.057	0.003
18	2.427	0.129	-1.905	0.248	0.976	0.952	59.043	0.078	0.005
19	1.870	0.137	-1.132	0.263	0.928	0.861	18.523	0.083	0.023
20	2.275	0.170	-2.130	0.326	0.967	0.934	42.589	0.103	0.007
21	2.679	0.187	-2.833	0.360	0.977	0.954	61.957	0.114	0.004
22	2.044	0.165	-1.268	0.318	0.917	0.841	15.894	0.101	0.028
23	2.452	0.181	-1.997	0.348	0.957	0.916	32.848	0.110	0.011

Stationary phase: C_{18} modified silica<br>Mobile phase consisted of methanol and water									
Compound number	R_M^0	σR_M^0	b	σb	R	R^2	F	s	p

1	1.65	0.148	-1.36	0.263	0.965	0.930	26.731	0.059	0.035
2	2.78	0.372	-3.31	0.663	0.962	0.926	25.000	0.148	0.038
3	2.75	0.210	-3.13	0.373	0.986	0.972	70.354	0.083	0.014
4	1.74	0.153	-1.85	0.272	0.979	0.958	46.088	0.061	0.021
5	2.29	0.245	-2.45	0.436	0.970	0.941	31.673	0.097	0.030
6	1.78	0.116	-1.45	0.207	0.980	0.961	49.407	0.046	0.020
7	1.87	0.174	-1.60	0.309	0.965	0.931	26.913	0.069	0.035
8	1.83	0.097	-1.71	0.173	0.990	0.980	97.178	0.039	0.010
9	1.86	0.120	-1.91	0.215	0.988	0.975	79.035	0.048	0.012
10	1.27	0.093	-1.04	0.166	0.976	0.952	39.501	0.037	0.024
11	1.72	0.150	-1.59	0.267	0.973	0.947	35.524	0.060	0.027
12	1.75	0.030	-1.75	0.053	0.999	0.998	1075.218	0.012	0.001
13	2.18	0.156	-2.47	0.278	0.988	0.975	78.933	0.062	0.012
14	2.45	0.176	-2.84	0.313	0.988	0.976	82.731	0.070	0.012
15	3.56	0.198	-4.21	0.352	0.993	0.986	143.007	0.079	0.007
16	2.62	0.188	-2.43	0.305	0.977	0.955	63.425	0.096	0.004
17	2.33	0.173	-2.03	0.280	0.973	0.946	52.350	0.089	0.005
18	2.54	0.257	-2.06	0.417	0.944	0.891	24.410	0.132	0.016
19	2.39	0.212	-1.78	0.321	0.969	0.939	30.550	0.072	0.031
20	2.85	0.360	-2.88	0.584	0.943	0.890	24.303	0.185	0.016
21	2.69	0.297	-2.55	0.481	0.950	0.903	28.053	0.152	0.013
22	2.79	0.168	-2.15	0.272	0.977	0.954	62.637	0.086	0.004
23	2.70	0.146	-1.98	0.236	0.979	0.959	69.995	0.075	0.004

Table S4. Correlation matrix of TLC chromatographic parameters.

	**R_M^0 C_{18}**	***b* C_{18}**	**C_0 C_{18}**	**mR_M C_{18}**	**PC1 C_{18}**	**R_M^0 C_8**	***b* C_8**	**C_0 C_8**	**mR_M C_8**	**PC1 C_8**
R_M^0 C_{18}	1.00	0.87	0.23	0.70	0.69	0.85	0.62	0.16	0.69	0.74
m C_{18}	0.87	1.00	0.67	0.26	0.24	0.76	0.80	0.45	0.27	0.35
C_0 C_{18}	0.23	0.67	1.00	0.49	0.50	0.26	0.66	0.69	0.46	0.38
mR_M C_{18}	0.70	0.26	0.49	1.00	1.00	0.59	0.10	0.30	0.96	0.95
PC_1 C_{18}	0.69	0.24	0.50	1.00	1.00	0.58	0.08	0.31	0.96	0.95
R_M^0 C_8	0.85	0.76	0.26	0.59	0.58	1.00	0.85	0.42	0.65	0.73
m C_8	0.62	0.80	0.66	0.10	0.08	0.85	1.00	0.75	0.15	0.26
C_0 C_8	0.16	0.45	0.69	0.30	0.31	0.42	0.75	1.00	0.31	0.21
mR_M C_8	0.69	0.27	0.46	0.96	0.96	0.65	0.15	0.31	1.00	0.99

PC_1 C_8	0.74	0.35	0.38	0.95	0.95	0.73	0.26	0.21	0.99	1.00

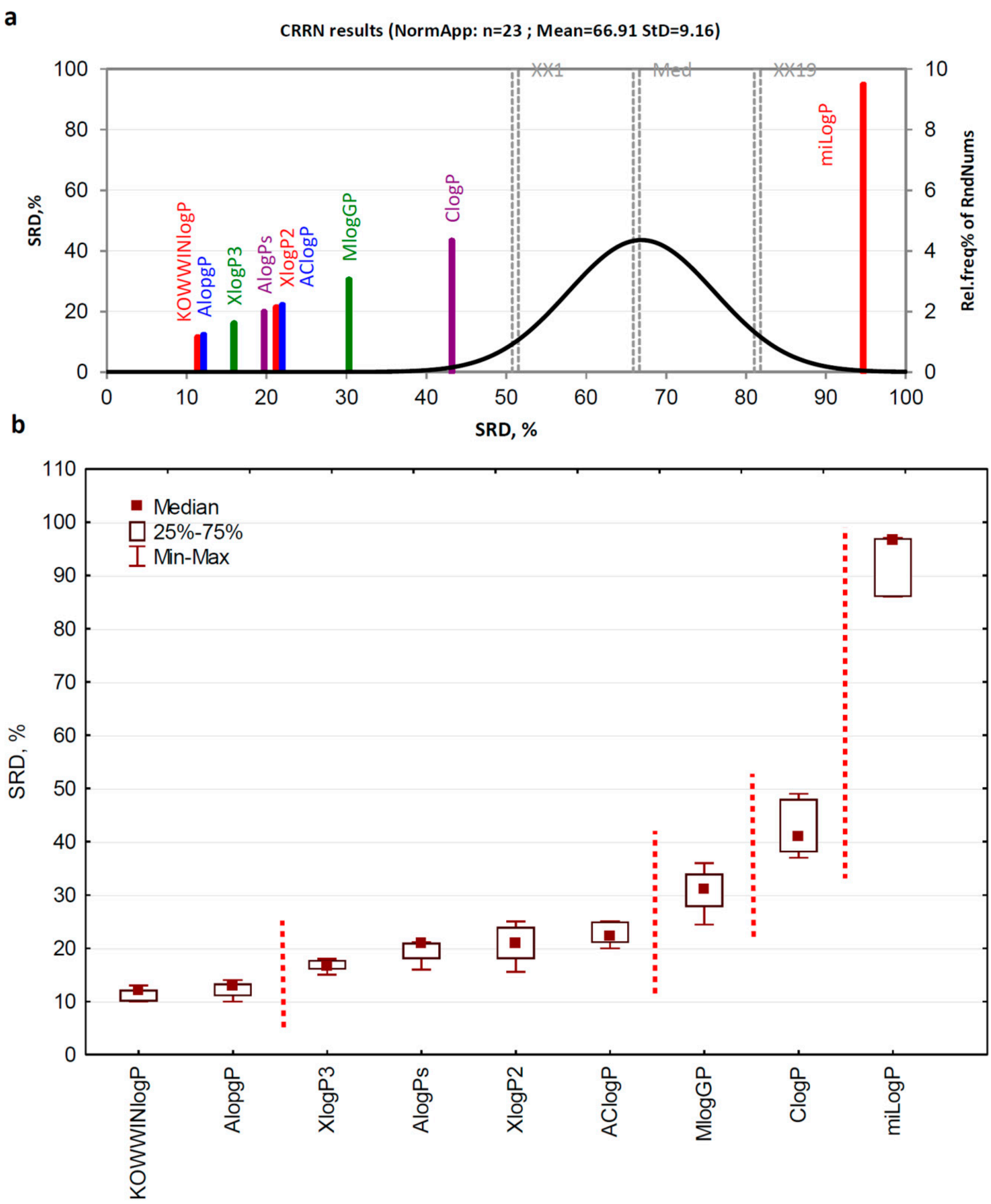

Figure S1. Ranking of computational methods for lipophilicity estimation. (**a**) sum of ranking differences-comparison of ranks by random numbers (SRD-CRRN) of interval scaled log*P* values; the SRD values are depicted on x and y-axis; (**b**) box and whisker plot of normalized SRD values obtained by the sevenfold cross-validation. Statistically significantly different methods ($p = 0.05$, tested by both the sign test and the Wilcoxon's matched pairs test) are separated by dashed lines.

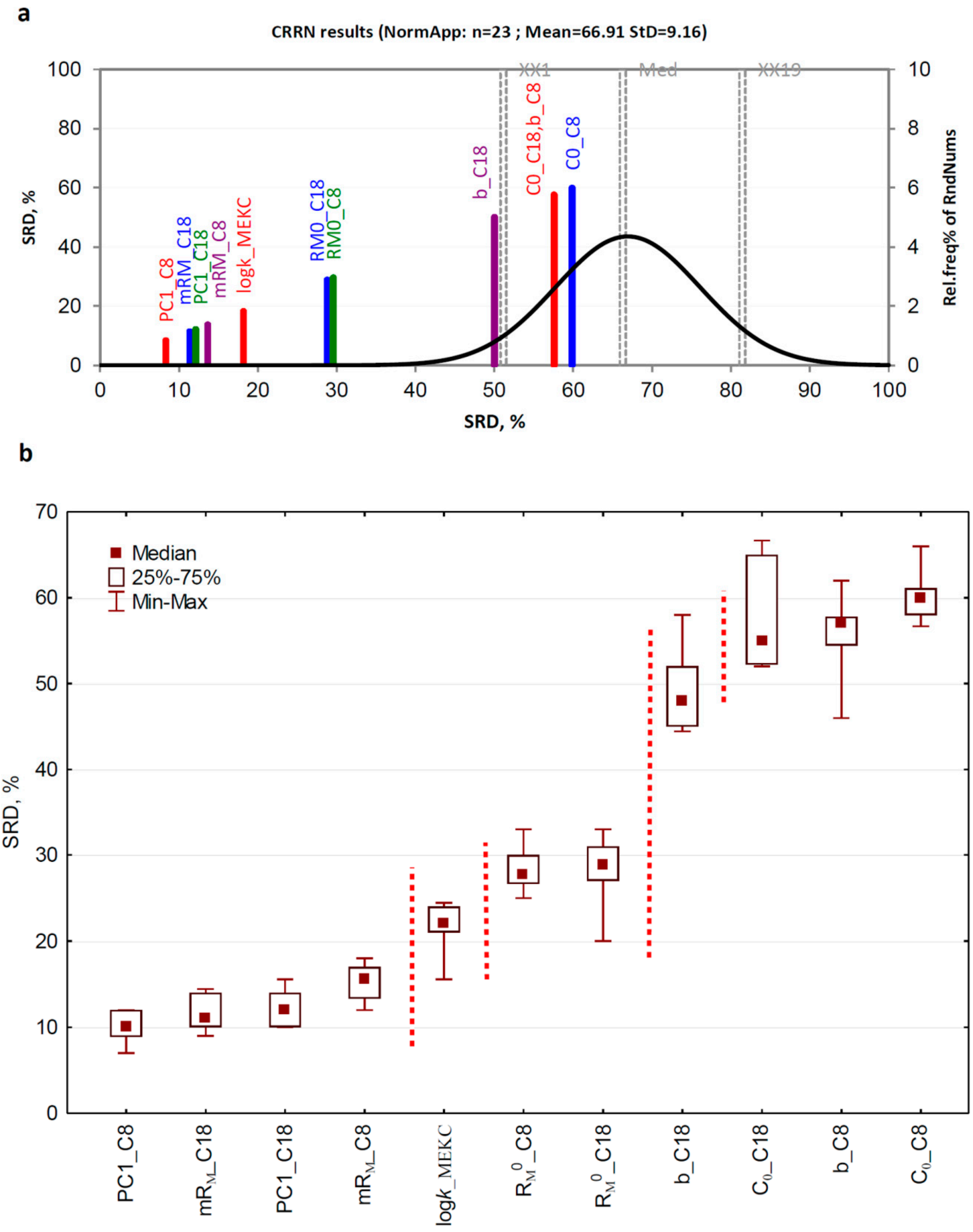

Figure S2. Ranking of chromatographic lipophilicity indexes. (**a**) SRD-CRRN of interval scaled chromatographic descriptors; the SRD values are depicted on x and y-axis; (**b**) box and whisker plot of normalized SRD values obtained by the sevenfold cross-validation. Statistically significantly different methods ($p = 0.05$, tested by both the sign test and the Wilcoxon's matched pairs test) are separated by dashed lines.

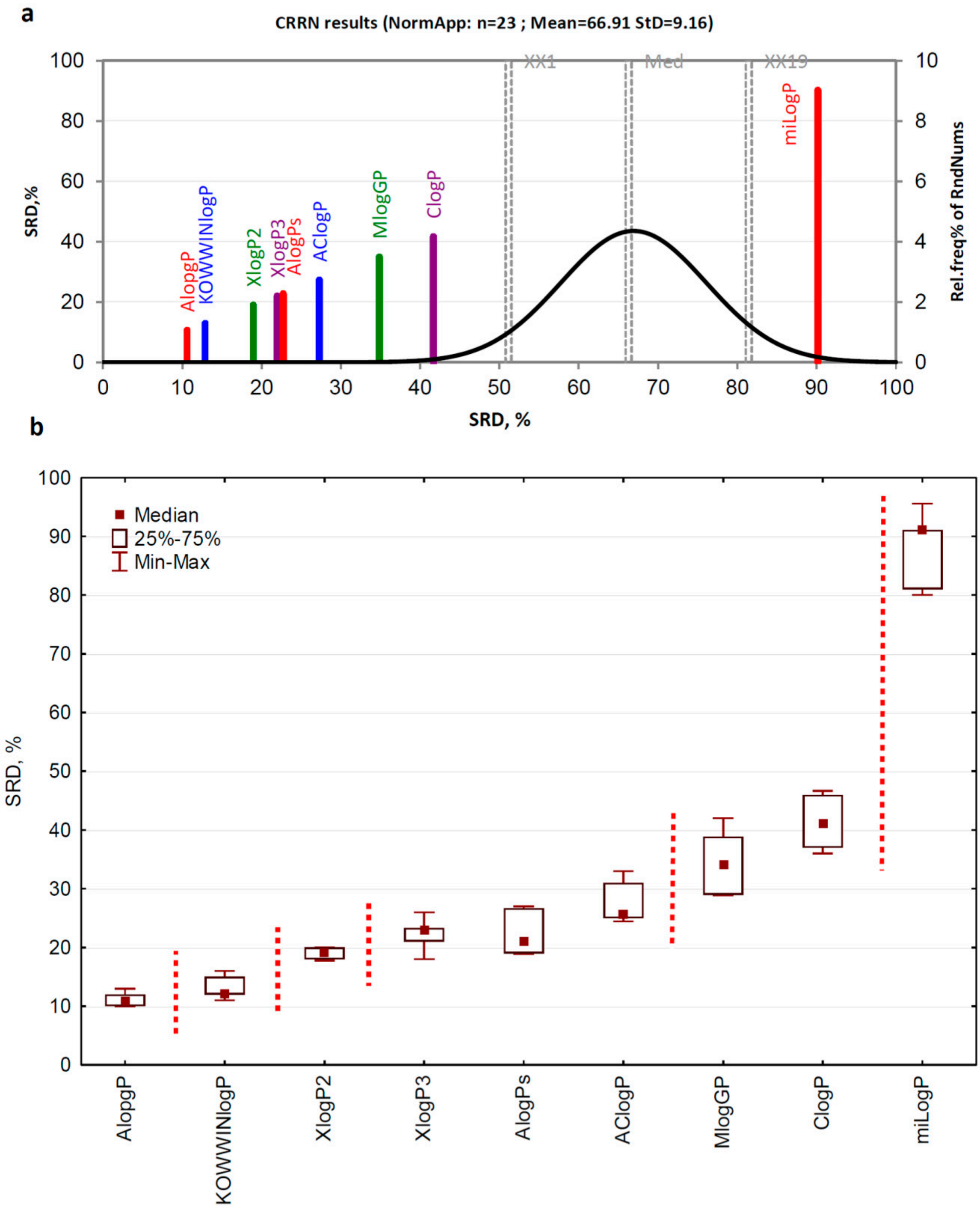

Figure S3. Ranking of computational methods for lipophilicity estimation. (**a**) SRD-CRRN of rank transformed log*P* values; the SRD values are depicted on x and y-axis; (**b**) box and whisker plot of normalized SRD values obtained by the sevenfold cross-validation. Statistically significantly different methods ($p = 0.05$, tested by both the sign test and the Wilcoxon's matched pairs test) are separated by dashed lines.

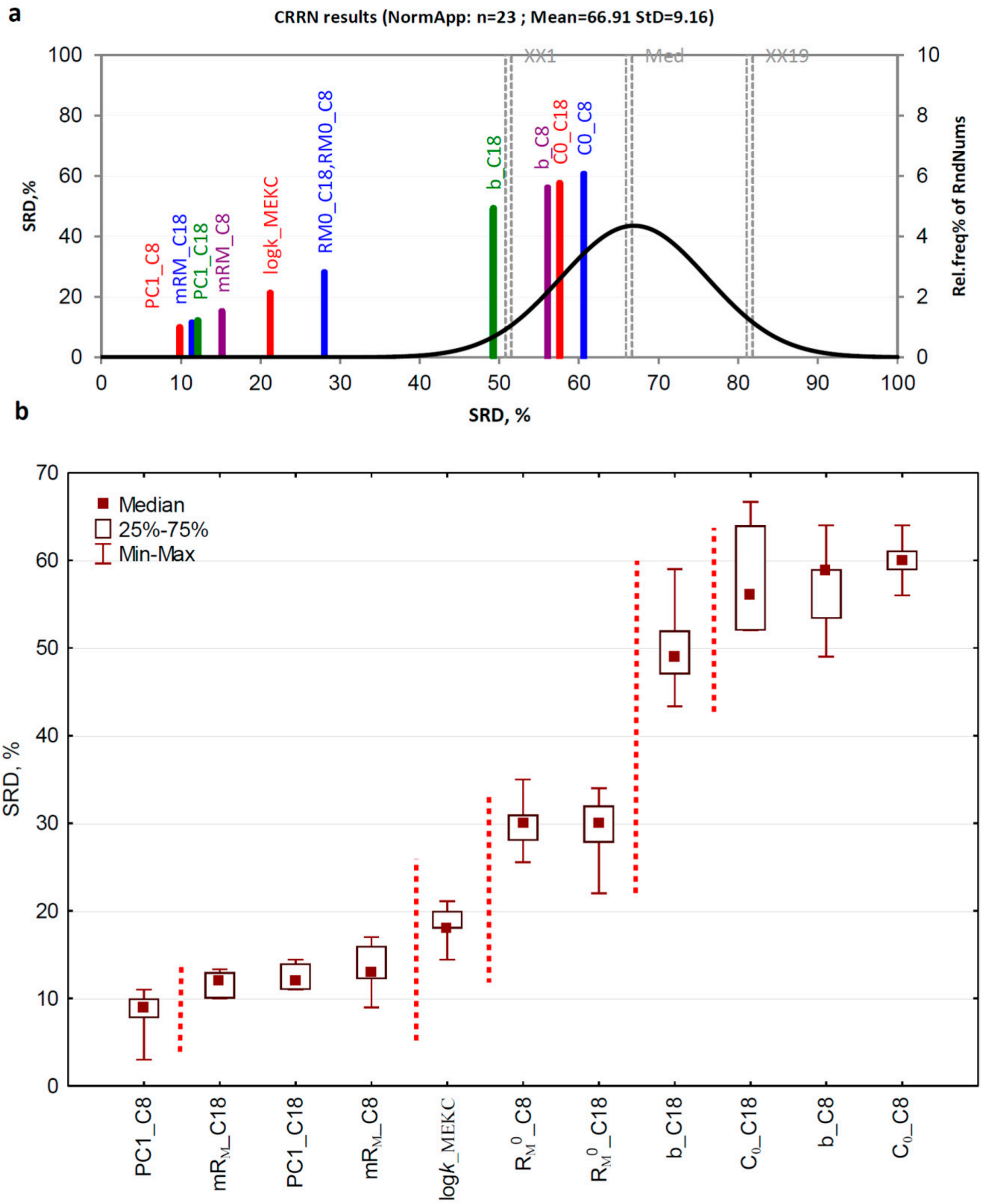

Figure S4. Ranking of chromatographic lipophilicity indexes. (**a**) SRD-CRRN of rank transformed chromatographic descriptors; the SRD values are depicted on x and y axes; (**b**) box and whisker plot of normalized SRD values obtained by the sevenfold cross-validation. Statistically significantly different methods (p = 0.05, tested by both the sign test and the Wilcoxon's matched pairs test) are separated by dashed lines.
